# Assessment of the Correlation between Appointment Scheduling and Patient Satisfaction in a Pediatric Dental Setup

**DOI:** 10.1155/2014/453237

**Published:** 2014-12-29

**Authors:** Amar N. Katre

**Affiliations:** Department of Pediatric and Preventive Dentistry, YMT Dental College & Hospital, Sector 4, Kharghar, Navi Mumbai-410210, India

## Abstract

*Introduction*. The practice of modern pediatric dentistry requires delivery of quality care in combination with adherence to excellent business as well as time management principles. A definite appointment schedule should be presented to the parents on the first or second appointment. More importantly, the prescribed schedule should be followed to the best of the professional abilities of the pediatric dentist. *Aims*. The aim of the study was to assess the co-relation between appointment scheduling and patient satisfaction in a pediatric dental setup with the objective of understanding the parameters related to appointment scheduling to increase patient satisfaction. *Method*. A total of 40 patients, who visited the Department of Pediatric and Preventive Dentistry, YMT Dental College & Hospital, for dental treatment were selected on a random basis. A questionnaire with a set of 6 questions with a rating scale of 1–5 to assess the patient satisfaction related to appointment scheduling was prepared. *Results*. A significant number of the patients were happy with the existing appointment scheduling system barring a few exceptions.

## 1. Introduction

The practice of modern pediatric dentistry requires delivery of quality care in combination with adherence to excellent business and management principles. Dentists traditionally trained in the art and science of dentistry must also become skilled in techniques of sound business management. Today's pediatric dental practitioner should be clinically astute and knowledgeable about consumer needs and demands, government regulations, third party participation, and so forth, aspects which may not have been taught in a dental school [1].

The primary responsibility of any dentist is to provide quality oral and dental health care to the patient. However, private practice, like any other business, must make a profit to survive. The dental practitioner thus should maintain a balance between patient care and business requirements while keeping moral, ethical, legal, and professional responsibilities in proper perspective [2, 3].

While maintaining this balance a pediatric dentist must provide a place where children can feel safe, loved, and well cared for, where parents can be educated about how to help their children have a lifetime of good oral health, where the staff know that they are an integral part of the practice and their opinions are counted for and where the community as a whole knows, respects, and appreciates the practice [4].

Pediatric dentistry is a unique specialty of dentistry as the patient, in the said case a child patient, cannot come alone to the dental clinic for his treatment but has to be brought by the parents who seek dental care for their child. Hence, the schedule of appointments and its related processes have to be discussed with the parents [5]. The parents of children could have different attitudes like overprotective, overindulgent, authoritarian, underaffectionate, rejecting, overanxious, and so forth. The manner in which the attitudes of parents (or patients) affect the appointment scheduling systems is a matter of further research [6].

To guide the parents into choosing the most suited appointment, the receptionist should be prepared with the relevant information to justify the time of scheduling [7]. Morning appointments are preferable in the young patients as they are fresh and active. The length of the appointment should be as short as possible (not greater than 30 minutes). The children should not be made to wait too long in the waiting room as they tend to get restless with passing time. Long waits for appointments decrease patient satisfaction [8]. A definite schedule, preferably designed in advance for individual patients, based on the magnitude of the dental problem and its subsequent management should be presented to the parents on the first or second appointment. More importantly, the prescribed schedule should be followed to the best of the professional abilities of the pediatric dentist [9].

Studies have been conducted over the world in an effort to devise and study patient appointment scheduling systems and the effectiveness of these systems in improving the quality of the practice as also the level of patient satisfaction. Taylor studied 639 patients who attended a practice whose surgeries are run on a mixed system of open access and by appointment. He concluded that the number of practices that run appointment systems has sharply increased; however such practices may have difficulty in fitting in urgent or extra consultations [10]. Campbell studied the relationship between (i) measures of how appointment systems work, (ii) patients' views of the arrangements for seeing their general practitioner, and (iii) practice self-referral rates to accident and emergency departments. These measures of appointment system operation correlated with patient dissatisfaction with the arrangements of seeing a doctor in their practice and with the perceived availability of a doctor to deal with nonurgent problems [11]. Aminabadi et al. studied a total of 450 children between 3 and 9 years of age to evaluate the age-specific effect of treatment duration on pediatric patient behavior. The study concluded that treatment duration may affect the behavior of pediatric patients parallel with chronological age and, thus, should be considered in the arrangement of the treatment plan. The findings of this study suggest appropriate pediatric behavior management should include thoughtful scheduling of appointments according to a treatment plan formulated with consideration of the effects of age and appointment length [12].

Thus appointment scheduling systems lie at the intersection of efficiency and timely access to health services. Timely access is important for realizing good medical outcomes. It is also an important determinant of patient satisfaction [13]. The goal of a well-designed appointment system is to deliver timely and convenient access to health services for all patients. Appointment systems also ensure smooth work flow, reduce crowding in waiting rooms, and allow health systems to honour patient and provider preferences while matching supply and demand. All these factors go a long way in promoting patient satisfaction, at the same time ensuring a high quality of dental care to the child [14].

With these objectives in mind, a study was carried outin the Department of Pediatric and Preventive Dentistry, YMT Dental College & Hospital, with the following aims and objectives.

*Aim*. To assess the co-relation between appointments scheduling and overall patient satisfaction in a pediatric dental setup.

*Objective*. To understand the parameters related to appointment scheduling and to incorporate necessary changes, if any, to increase patient satisfaction.

## 2. Materials and Methods

The study was carried out in the Department of Pedodontics and Preventive Dentistry, YMT Dental College & Hospital, Kharghar, Navi Mumbai. A cross sectional closed ended questionnaire study was designed. A total of 40 patients, ages ranging from 8 to 12 years, who visited the Department of Pediatric and Preventive Dentistry, YMT Dental College & Hospital, for dental treatment were selected on a random basis. Only those patients and their parents were included who had reached or were in the process of reaching the completion of their proposed treatment. To further standardise the process of appointment scheduling and related parameters of patient satisfaction and eliminate bias, only those patients under treatment by postgraduate students (already have completed graduation and hence are registered dental practitioners) were included as appointments for the said patients were given by the postgraduates themselves and not by the support staff of the department. Necessary permissions were obtained from the institution and consent of the participants was taken prior to the start of the study.

A questionnaire with a set of 6 questions with a Likert rating scale of (1)–(5) (the appendix) to assess the patient satisfaction related to appointment scheduling was prepared. The questionnaire was pretested for face and content validity. The parents of the selected patients were interviewed as the patients assessed belonged to the pediatric age group who could not possibly analyse and fill the questionnaire. The parents of the selected children were explained the meaning of the questions and the criteria of rating. Subsequently, they were asked to fill in the questionnaire and instructed to give the most accurate rating to the prescribed set of questions. Further, they were instructed to fill out the questionnaire in isolation of the operator or another parent to avoid any bias in rating the answers to the questions. The parents were instructed to abstain from writing their names or putting their signatures on the questionnaire forms in an effort to protect their identity. The parents were encouraged to give their valuable comments/suggestions for improving the services provided by the department (in relation to appointment scheduling) as regards (Q. 1) to (Q. 5). The filled questionnaire was collected by the researcher.

The data so collected was tabulated and statistically evaluated. Frequency distribution and percentage analysis was done. Spearman's correlation coefficient was used for correlating the individual experience with the overall patient satisfaction.

## 3. Results

Table 1 shows the ratings of the questionnaires that were obtained from the parents of the patients. The average rating for (Q. 1) that was related to the procedure of appointment procurement was 3.675 which indicates that the majority of the patients were neutral (neither happy nor unhappy), with inclination towards being happy with the procedure. For (Q. 2) that was related to the number of appointments for the procedure, the average rating was 3.45 which indicated that majority of the patients were inclined towards a neutral (neither happy nor unhappy) rating. For (Q. 3) that was related to the duration of each appointment, the rating of 3.725 indicated that majority of the patients were happy with the same. For (Q. 4) that was related to the following of the prescribed schedule, the rating was 3.675 that indicated that again most patients were happy with this parameter. For (Q. 5) that was related to waiting experience, a rating of 3.5 was indicative of neutral to happy feeling of the patients towards this parameter. For (Q. 6) that was related to overall satisfaction, a rating of 3.775 indicated that there was a degree of happiness towards the entire process of appointment systems.

Table 2 shows the distribution of patient satisfaction related to the parameters of questionnaire.

**Figure 1:**
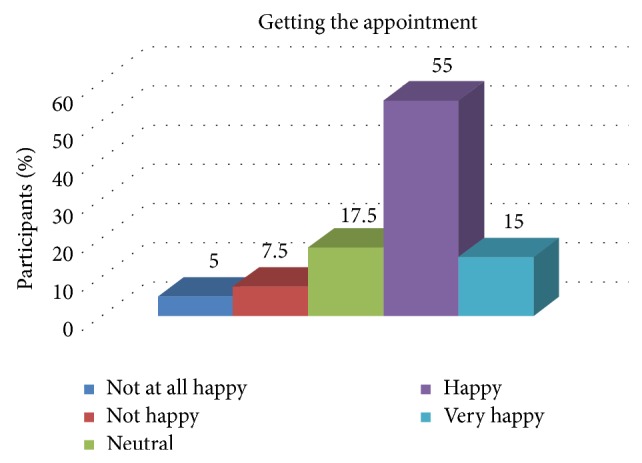
Distribution of patient satisfaction related to getting the appointment.

Table 3 depicts the association of the overall experience of the patient with the experience of getting the appointment. The same is depicted in Figure 1. There was a statistically significant association between the experience of the patient and the experience of getting the appointment.

**Figure 2:**
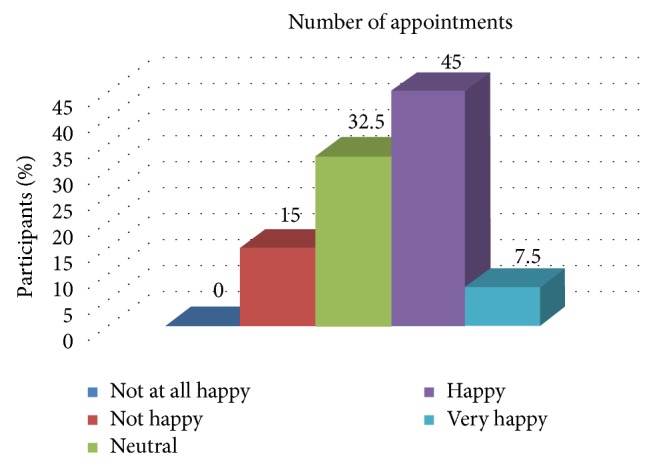
Distribution of patient satisfaction related to the number of the appointments.

Table 4 and Figure 2 show the association of the overall experience of the patient with the number of appointments. There was a statistically significant association between the experience of the patient and the number of appointments.

**Figure 3:**
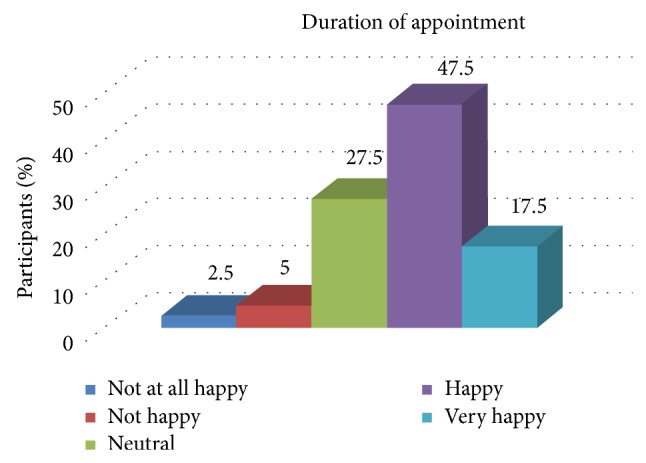
Distribution of patient satisfaction related to the duration of the appointment.

Table 5 and Figure 3 show the association of the overall experience of the patient with the duration of the appointments. There was a statistically significant association between the experience of the patient and the duration of appointments.

**Figure 4:**
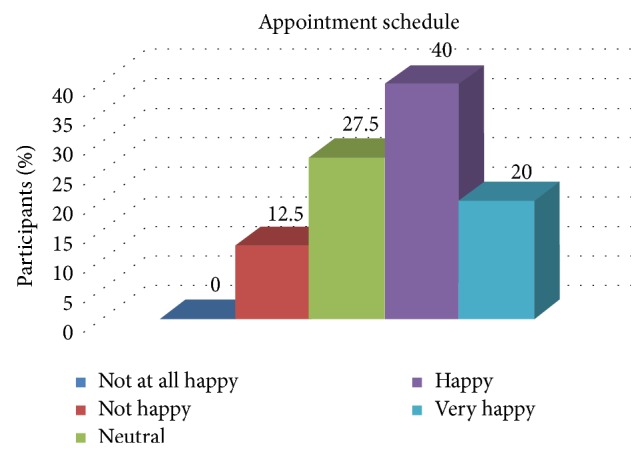
Distribution of patient satisfaction related to the appointment schedule.

Table 6 depicts the association of the overall experience of the patient with the appointment schedule. The same is shown in Figure 4. There was a statistically significant association between the experience of the patient and the appointment schedule.

**Figure 5:**
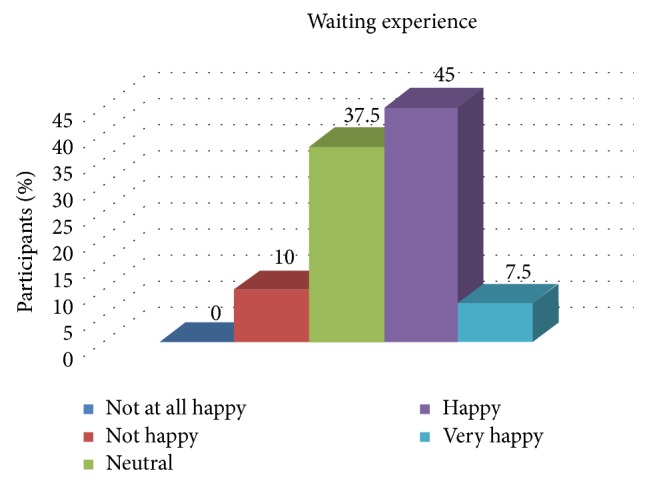
Distribution of patient satisfaction related to the waiting experience.

Table 7 and Figure 5 show the association of the overall experience of the patient with the waiting experience. There was a statistically significant association between the experience of the patient and the waiting experience.

**Figure 6:**
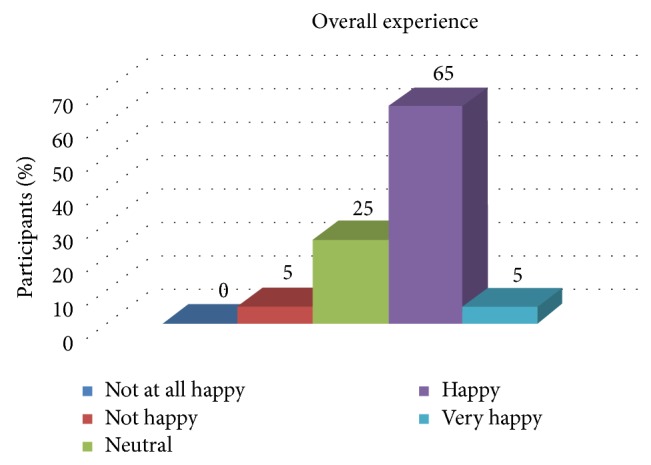
Distribution of patient satisfaction related to the overall experience.

Figure 6 shows the distribution of patient satisfaction related to the overall experience.

## 4. Discussion

The scheduling of appointments is of utmost importance in a pediatric dental setup. This is mandatory to deliver quality health care in an effective and efficient manner to the child patients [1]. In addition, the scheduling of an appointment and the following of the schedule for a child patient is directly correlated to the behavior management plan parallel with chronological age and, thus, should be considered while devising and presenting the treatment plan [14]. The world over, various systems have been devised and studied to improve the appointment scheduling systems so as to render quality treatment to patients. Most of these systems have been studied in reference to the various branches of medicine, but there is a paucity of literature related to pediatric dentistry. Most of these systems have concluded that though appointment scheduling does help to improve the quality of health care delivery, not all the systems may be patient friendly and patient satisfaction with these systems varies [12, 13, 15, 16].

In our study we found that a majority of the patients were happy with the existent appointment system that was being followed in the Department of Pediatric Dentistry, YMT Dental College & Hospital, where the present study was carried out. However, there was a section of the parents who were not at all happy with the existent appointment system, especially related to the process of procuring the appointment and the duration of each appointment (Tables 2, 3, and 5). This unhappiness could possibly be related to not getting the appointments of their choice, which may not be possible every time in a pediatric dental setup owing especially to the fact that the timings of the dental hospital (where the study was conducted) entail the child to miss his school. The unhappiness could also be linked to operator variation, especially related to duration of the appointments, as each operator may not possess the same competency as another operator, who may be more efficient at time management. Also, in a typical pediatric dental setup, the duration of the appointment cannot be kept above a particular duration (ideal not greater than 30 min) which could be resented by certain patients, considering the fact that they may have travelled a considerable distance to reach the hospital. It is also important to note that a number of patients also were unhappy with the following of the appointment schedule (Tables 2 and 6), which could be totally related to the operator being less competent or due to the absence of a particular operator on the day the appointment was scheduled.

Many patients chose to remain neutral on the issues of number of appointments, the duration of the appointments, the following of the schedule of appointments, and the waiting experience (Table 2). This indicated that if the department can incorporate improvements in all aspects of the appointment scheduling systems it will definitely help improve the rating of the overall experience and could elevate this class of patients from the neutral category to the happy or very happy category. It was also observed that the difference between the happy patients and neutral patients related to parameters like number of appointments, appointment schedule implementation, and waiting experience was not very high (Table 2).

In our study, an assessment of the descriptions provided by the parents under the subheading of “Any other comments/suggestions to improve the services provided as regards appointment systems” showed that most of the parents were most concerned about the number of appointments and the average waiting time. Some parents suggested that improving the infrastructure in the children's waiting area and reducing the number of appointments would attract more patients to the hospital.

It is probably these areas on which the department should toil so that the level of patient satisfaction could be increased. The happy overall experience could be attributed to the fact that the appointments were being given by postgraduates themselves under the supervision of the staff. Also, the treatment was being done by individuals who had received adequate training in the subject of pediatric dentistry. In addition, the treatment plan for each patient was thoroughly discussed and approved by the staff, thereby leading to more efficient delivery and completion of the treatment. However, reduction of the number of appointments to a bare minimum may not be possible given the differences in the nature of oral and dental diseases and differences in the behavioural temperament of each child.

The department should aim at incorporating changes in the existing appointment scheduling to convert that category of patients who were unhappy or neutral with the appointment scheduling system to the happy or extremely happy category. Standardization of the procedure, increasing the competency of all the operators, minimizing the waiting period, extending the working hours so that the child may not miss school, and improving the infrastructure of the department, especially the waiting area, are the areas where the department can concentrate on achieving the above mentioned aim. Periodic reassessment will also enable maintaining the standards of the appointment scheduling system. More research on appointment scheduling using different parameters like age, gender, occupation of parents, and so forth and their correlation with parent satisfaction needs to be conducted.

The real challenge would be to provide maximum possible levels of satisfaction to the patients in relation to all aspects of the health care delivery system.

## Figures and Tables

**Table 1:** Likert ratings for the questionnaire (40 patients).

Q. 1	Q. 2	Q. 3	Q. 4	Q. 5	Q. 6
4	3	3	4	3	4
5	5	5	5	5	5
4	4	4	5	4	4
3	4	3	3	3	4
4	4	4	3	4	4
4	3	4	4	4	4
4	5	4	4	4	4
4	4	4	4	3	4
4	4	4	5	3	4
4	5	5	4	4	5
4	4	4	4	4	4
4	4	4	4	4	4
5	4	5	5	4	4
1	4	4	4	3	4
4	4	3	3	2	3
4	4	5	4	4	4
5	4	5	4	4	4
4	4	4	5	3	4
4	3	4	3	3	3
2	2	3	3	3	3
4	4	3	3	4	4
3	4	4	4	3	4
2	2	3	2	2	3
3	2	2	2	2	3
3	4	3	4	3	3
4	3	4	3	4	3
4	3	4	3	4	3
4	3	4	4	4	4
4	3	4	4	4	4
3	3	4	4	4	4
3	2	3	2	4	4
4	2	2	4	3	4
4	3	4	3	3	4
4	3	3	3	4	4
3	3	3	5	3	3
5	3	3	3	3	4
5	4	5	5	5	4
5	4	5	5	5	5
2	3	4	2	3	3
1	2	1	2	2	2

3.675	3.45	3.725	3.675	3.5	3.775

**Table 2:** % distribution of patient satisfaction related to the parameters of the questionnaire.

	Not at all happy	Not happy	Neutral	Happy	Very happy
Getting the appointment	5%	7.5%	17.5%	55%	15%
Number of appointments	0%	15%	32.5%	45%	7.5%
Duration of appointment	2.5%	5%	27.5%	47.5%	17.5%
Appointment schedule	0%	12.5%	27.5%	40%	20%
Waiting experience	0%	10%	37.5%	45%	7.5%
Overall experience	0%	5%	25%	65%	7.5%

**Table 3:** Association of the overall experience of the patient with the experience of getting the appointment.

	Not at all happy	Not happy	Neutral	Happy	Very happy
Getting the appointment	5%	7.5%	17.5%	55%	15%
Overall experience	0%	5%	25%	65%	5%

Pearson's chi square	38.145				
Spearman's rho	0.559				
*P* value	0.000				

**Table 4:** Association of the overall experience of the patient with the number of appointments.

	Not at all happy	Not happy	Neutral	Happy	Very happy
Number of appointments	0%	15%	32.5%	45%	7.5%
Overall experience	0%	5%	25%	65%	5%

Pearson's chi square	28.110				
Spearman's rho	0.562				
*P* value	0.001				

**Table 5:** Association of the overall experience of the patient with the duration of the appointments.

	Not at all happy	Not happy	Neutral	Happy	Very happy
Duration of appointment	2.5%	5%	27.5%	47.5%	17.5%
Overall experience	0%	5%	25%	65%	5%

Pearson's chi square	59.141				
Spearman's rho	0.546				
*P* value	0.000				

**Table 6:** Association of the overall experience of the patient with the appointment schedule.

	Not at all happy	Not happy	Neutral	Happy	Very happy
Appointment schedule	0%	12.5%	27.5%	40%	20%
Overall experience	0%	5%	25%	65%	5%

Pearson's chi square	21.537				
Spearman's rho	0.555				
*P* value	0.01				

**Table 7:** Association of the overall experience of the patient with the waiting experience.

	Not at all happy	Not happy	Neutral	Happy	Very happy
Waiting experience	0%	10%	37.5%	45%	7.5%
Overall experience	0%	5%	25%	65%	5%

Pearson's chi square	35.074				
Spearman's rho	0.608				
*P* value	0.000				

## Appendix

### Appointment Scheduling Questionnaire

Please read the underlying questions and give the most accurate rating as per the rating scale described.

*Rating Scale*

(1) Not at all happy
(2) Not happy
(3) Neutral
(4) Happy
(5) Very Happy

(Q. 1) How was your experience in getting the appointment?
Rating:
(Q. 2) Were you satisfied with the number of appointments for the prescribed treatment?
Rating:
(Q. 3) Were you satisfied with the duration of the each appointment?
Rating:
(Q. 4) Was the appointment schedule given to you followed while doing the treatment?
Rating:
(Q. 5) How was experience related to the average waiting time?
Rating:
(Q. 6) How was your overall experience?
Rating:

Any other comments/suggestions to improve the services provided as regards appointment systems.
